# The Editor’s Desk

**Published:** 1916-10

**Authors:** C. N. Johnson


### THE EDITOR'S DESK.

### My Prayer.

I pray that I shall be patient—patient with the mistakes, the foibles, and even the sins of others. I pray that if I am wronged I shall be patient with the wrong-doer; that I shall count all the causes which led to the wrong; that I shall search faithfully to see if I may not in some manner have contributed to the wrong. I pray that my patience extends not to myself in case I do wrong to others, that the righteous indignation of my whole soul shall rise up within me to accuse me in all bitterness. I pray that I may have charity—not the blatant kind that builds edifices and dedicates them with blare of trumpet; but the kind that goes down deep into the aching hearts of humanity and forgives a false or wayward step. I pray that the sins of the fathers be not meted out to the sons, that the dismal heritage of disease and pain be banished from the generations of men. I pray that the pangs of hunger be not inflicted on the children of the poor, nor the pride of possession be the chief portion of the rich. I pray for the virtue of humility—not the humility of self-consciousness, but the humility of sacrifice and service. I pray for the blessing of work—not the agonized toil of helpless women and children through greed of gain, but the glorious privilege of doing for the sake of doing, of adding to the myriad means of human thrift and happiness and increasing day by day the material and moral welfare of the world. I pray for hope, that blessed balm of the human soul; for love, that elixir of the human heart. I pray for the rugged path of truth, the high hill of justice, and the boundless atmosphere of liberty. I pray for stress and storm, for heat and cold, for light and darkness—for the constant change which holds within it the germ of virile growth. I pray for reverses to try the temper of my mind, that I may better bear the favors of fortune when they come. I pray for breadth of vision, for height of aspiration, and for depth of discernment. I pray or purity of thought—the mentor of our every act, the guardian angel of our deeds, and the star which keeps our faces forever turned toward the light—above all I pray for purity of thought. I pray for happiness, not the false and spurious happiness which grows in a heaven of lure and lust, but the happiness which falls as the gentle dew from the vine of virtue—the happiness which comes from doing good and being kind. I pray that I may be like other men, with the same hopes, the same desires, and the same trials—that there be no aloofness in my life. I pray that I may live and labor with my fellow-man. I pray that I may be blessed with friends and companions; friendship the flower, and companionship the fruit of earthly experience. I pray that I may love and be loved as long as I live, that I may bask in the sweet savor of that rarest plant of human life, and close my eyes at last to the soothing melody of loving lips.

And in praying for all of this I make the prayer to myself, knowing full well that the answer lies for the most part with the one who prays.—Dr. C. N. Johnson, *Dental Review.*

